# Minimally Invasive Cutting and Suture Technique for the Management of Caudal Septal Deviation

**DOI:** 10.7759/cureus.78104

**Published:** 2025-01-27

**Authors:** Tomohisa Hirai, Tsutomu Ueda, Takashi Ishino, Takao Hamamoto, Sachio Takeno, Takehiro Sera

**Affiliations:** 1 Otolaryngology, Hiroshima Prefectural Hospital, Hiroshima, JPN; 2 Otolaryngology - Head and Neck Surgery, Hiroshima University, Hiroshima, JPN

**Keywords:** caudal end deviation, cutting and suture technique, minimally invasive cutting and suture technique, nasal tip height, n/w ratio

## Abstract

Objective: Surgery for caudal septal deviations is challenging due to cartilage shape memory, the need for adequate nasal tip and dorsal septal support, and long-term healing effects. Here, we report a new surgical method for caudal septal deviations called the minimally invasive cutting and suture technique (MICST). Although similar to the cutting and suture technique, MICST preserves the tissue around the posterior septal angle by maintaining the continuity of the nasal septum cartilage from the keystone area to the anterior nasal spine. The direction of the cutting line is parallel to the dorsal line of the nose, allowing it to release excess pressure from both dorsal and caudal deviations.

Methods: A total of 45 patients underwent MICST between September 2022 and August 2023. We analyzed data collected preoperatively and 3 months postoperatively to compare the cross-sectional area ratios of the convex side (narrower) and the concave side (wider), known as the N/W ratio and nasal tip height (using computed tomography), visual analog scale, and operative time with those of 45 patients who underwent the standard cutting and suture technique (CST) between September 2020 and July 2022.

Results: Significant differences were not observed in preoperative and postoperative N/W ratio, and visual analog scale scores between the MICST and CST groups. The proportion of cases in which the nasal tip decreased by ≥3 mm was higher in the CST group, and the operative time was shorter in the MICST group.

Conclusion: The improvement of nasal obstruction using MICST is equivalent to that of CST. Compared to conventional methods, MICST results in the more conservative treatment of the nasal septal cartilage, carries a smaller risk of external nasal deformity, allows correction of dorsal deviation, and does not require a batten graft. The procedure is straightforward and can be performed in a short time.

## Introduction

The nasal septum is an osteocartilaginous wall that divides the nose into two nasal cavities. A minor asymptomatic deviation of the septum is considered a normal developmental variation found in most of the population. For some patients, the degree of deviation may cause obstruction and affect nasal airflow [1]. Caudal septal deviations cause more functional problems than other types of septal deviations. The severity of a caudal septal deformity or deviation can result in varying levels of nasal obstruction [2]. An excessively long caudal septum is the primary cause of caudal end deviation (CED) of the nasal septum [3]. Latent deviation of the caudal end may become apparent postoperatively if the CED is not recognized and corrected as a standard septal deviation. Therefore, clinicians should evaluate CED and correct it appropriately [4,5]. However, surgery for CED is considerably more challenging than that for standard septal deviation and can also cause external nasal deformities, such as saddle nose or nasal tip (NT) dropping [6].

Here, we report a novel surgical method, the "minimally invasive cutting and suture technique” (MICST), which is a surgical technique developed based on CST and aims to maintain the stability of the external nose by performing it more conservatively.

## Materials and methods

Hospitalized patients who underwent MICST to alleviate nasal obstruction in our department from April 2020 to October 2023 were included in the study. We excluded patients with severe external nasal deformities. The study was designed to have an equal number of patients receiving CST or MICST. CST was performed from April 2020 to July 2022, and MICST from August 2022 to October 2023.

All surgeries were performed by a single surgeon (TH). Written informed consent was obtained from all participants before their inclusion in the study. This study was approved by the Ethics Committee of Hiroshima Prefectural Hospital (R5-23-3, 2023) and was conducted in accordance with the Helsinki Declaration.

The follow-up period at our hospital was defined from the date of surgery to 3 months postoperatively. For subjective evaluation, we compared the nasal obstruction status using the visual analog scale (VAS) preoperatively and 3 months postoperatively, with scores ranging from 0 (no nasal obstruction) to 10 (complete nasal obstruction). Subsequently, patients were followed up at the referring clinic at 6 months and then annually. For objective evaluation, we analyzed computed tomography (CT) data to compare the cross-sectional area ratios of the convex side (narrower) and the concave side (wider), known as the N/W ratio, both preoperatively and 3 months postoperatively [5]. The N/W ratio is defined as the ratio of the cross-sectional areas at the nostril entrance. To determine this, a coronal plane intersecting the mid-sagittal plane at 90° was used, incorporating two landmarks: the anterior nasal spine (ANS) and the anterior tip of the nasal bone, analyzed using SYNAPSE VINCENT® software (Fujifilm Medical Co., Ltd., Tokyo, Japan) [5].

**Figure 1:**
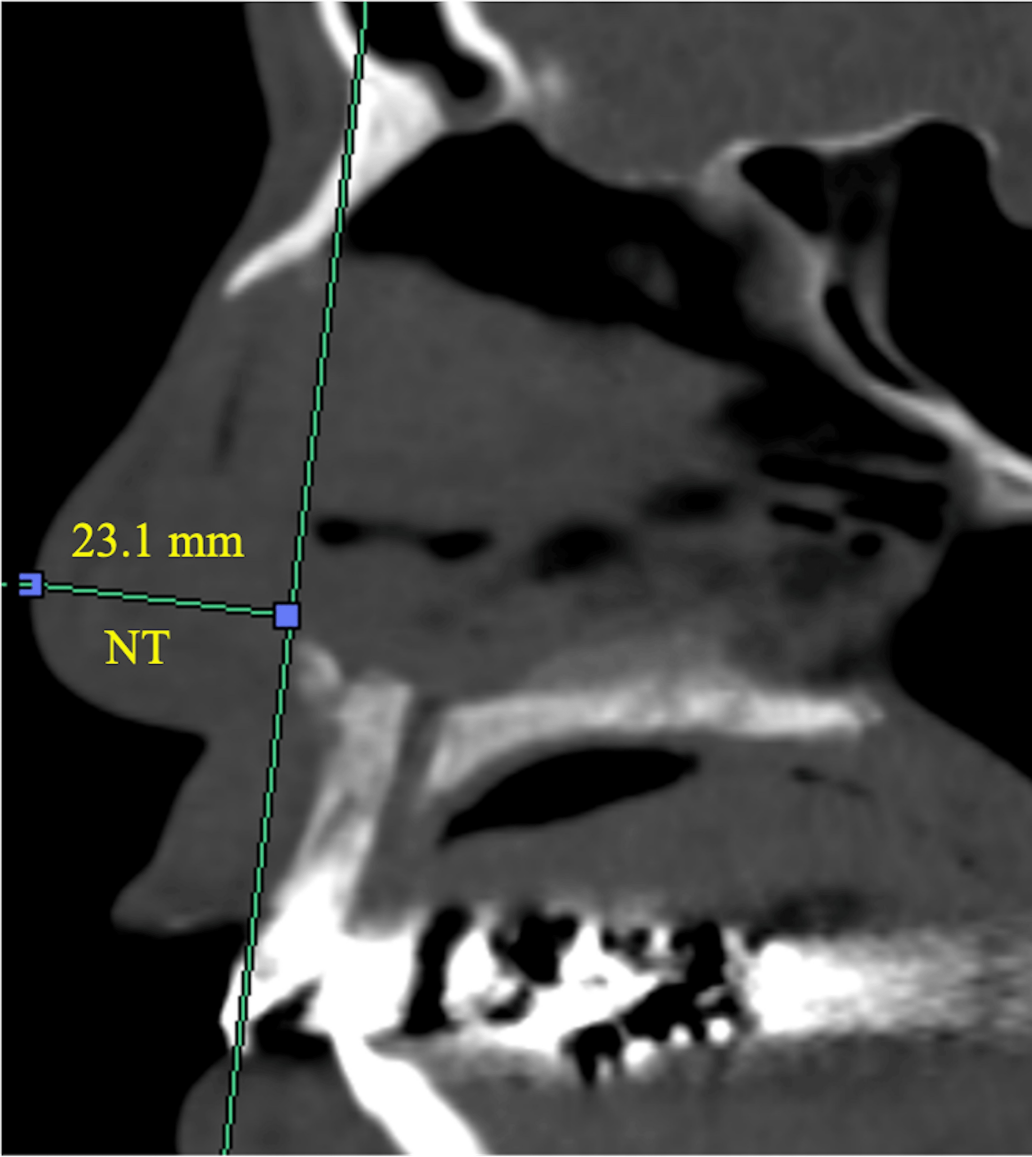
NT measurement The NT height was measured as follows: a vertical line was drawn from the line connecting the nasion (the midpoint of the frontal bone at the top of the nose) and the maxillary central incisor (the upper front tooth) to the NT, and the distance was measured. NT: nasal tip

To assess the effect of MICST on external nasal morphology, we measured the NT height by drawing a vertical line from the line connecting the nasion and maxillary central incisor to the NT, and the distance was measured [7]. Figure 1 shows a concrete NT measurement of 23.1 mm. We also examined the proportion of cases in which the NT decreased by more than 3 mm postoperatively. No prior studies have investigated how such a decrease in NT is perceived as an external change. In this study, the average NT was approximately 23 mm. We established a cutoff value of 3 mm, hypothesizing that a decrease exceeding 10% may be recognized as a change in appearance. 

The VAS scores, N/W ratio, NT, and operative time were compared between patients who underwent MICST and those who underwent CST at our department from April 2020 to October 2023. Patient demographics and CT data were analyzed using the Student's t-test, while the Mann-Whitney test was used to analyze the proportion of cases in which the NT decreased by ≥3 mm. This study did not aim to establish statistical equivalence and discuss the clinical relevance of observed differences, such as changes in NT.

**Figure 2:**
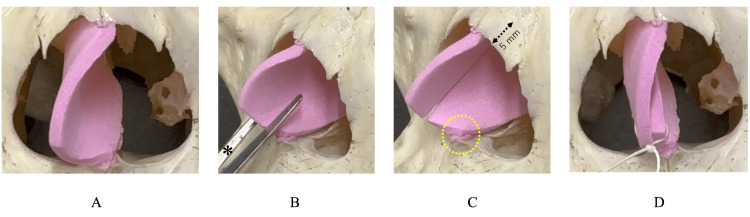
Schematic diagram of the MICST A. Preoperative image of the caudal end deviation. B. Nasal septal cartilage cut from the caudal end using scissors, with its direction parallel to the dorsal line of the nose. C. Anterior part of the nasal septal cartilage preserved by approximately 5 mm. D. Overlapping upper and lower portions of the cartilage sutured using a single stitch. Dotted circle: range of preserving connective tissues surrounding the anterior nasal spine; dashed bidirectional arrow: range of preservation of the anterior nasal septal cartilage (approximately 5 mm). MICST: minimally invasive cutting and suture technique

**Figure 3:**
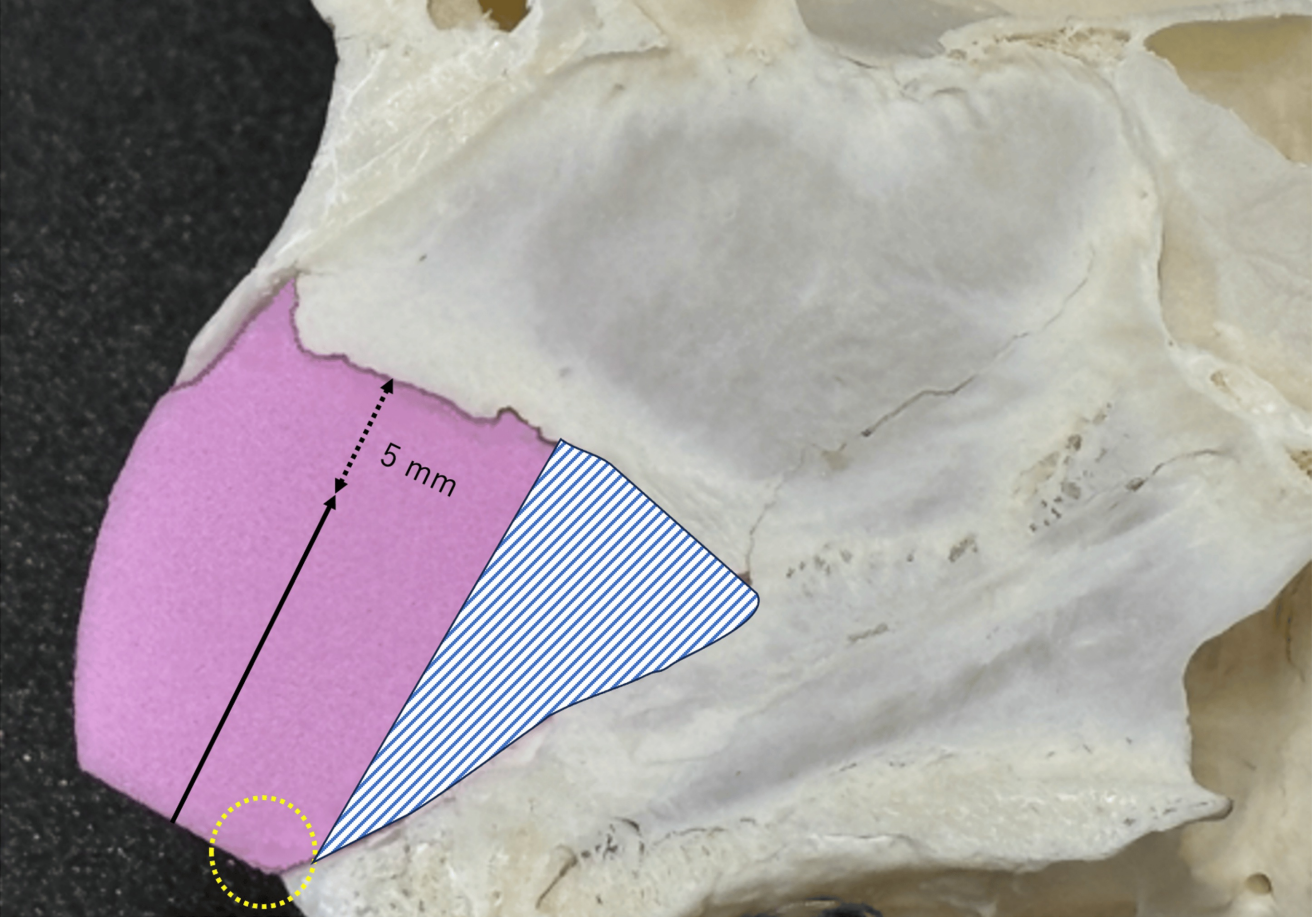
Schematic of the portions requiring preservation Solid arrow: cutting line parallel to the dorsal line of the nose initiated from the center of the caudal end; dashed bidirectional arrow: range of preservation of the anterior nasal septum (approximately 5 mm); dotted circle: range of preserving connective tissues surrounding the anterior nasal spine; hatched areas: portion of the lower part of nasal septal cartilage that was resected.

**Figure 4:**
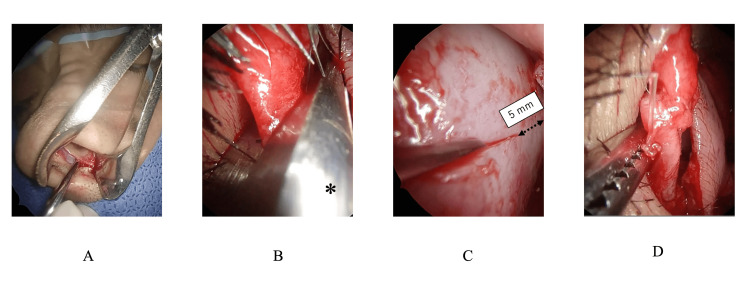
Operative findings of the MICST A. Caudal end exposed through a hemitransfixion approach. B. Cutting initiated from the center of the caudal end, with its direction parallel to the dorsal line of the nose. C. Posterior end of the nasal septal cartilage preserved by approximately 5 mm. D. Overlapping upper and lower portions of the cartilage sutured using a single stitch. *scissors; dashed bidirectional arrow: range of preservation of the anterior nasal septal cartilage (approximately 5 mm). MICST: Minimally invasive cutting and suture technique

Figure 2 and Video 1 present the schematic diagrams of the MICST. Figure 3 illustrates the portions to be preserved. Figures 4 and Video 2 present the operative findings of the MICST.

**Video 1:** The schematic diagrams described elements of the MICST MICST: minimally invasive cutting and suture technique; ANS: anterior nasal spine

**Video 2:** Operative findings summarizing the main points of the MICST MICST: Minimally invasive cutting and suture technique

Lidocaine (2%) with 1:80,000 epinephrine was infiltrated, and a hemitransfixion incision was made 1-2 mm behind the anterior margin on the concave side of the anterior septum using a No. 15 blade under anterior rhinoscopy. The mucoperichondrial flap was elevated by sharp dissection on both sides of the septum, exposing the caudal end (Figure 2A). The following procedures were performed under endoscopy. After we elevated of the mucoperichondrial flaps on both sides to the extent possible, the cartilaginous septum was released from the bony septum. Next, the perpendicular plate of the ethmoid, vomer, the lower part of nasal septum cartilage and maxillary crest, including the premaxillary wing, were resected, while preserving the connective tissues surrounding the anterior nasal spine (ANS) and posterior septal angle (PSA) (Figures 3C, 4). The cutting of the cartilaginous septum began from the center of the caudal end, parallel to the dorsal line of the nose (Figure 2B, 3B). To ensure nasal septal continuity, the posterior end of the nasal septal cartilage was maintained at approximately 5 mm (Figure 2C, 4C). If you cut the part of the cartilage to be preserved (the 5mm preservation part) it will be almost the same as CST, and it may reduce the height of the nasal tip and shorten the caudal septal length, resulting in saddle nose deformity. The overlapping upper and lower portions of the cartilage were sutured together with a single 4-0 polydioxanone stitch (Figure 2D, 4D). Batten graft fixation was not required. Penetrating sutures were placed in the mucosa-cartilaginous mucosa to prevent the formation of a nasal septal hematoma. Silicone plates were affixed to both sides of the nasal septum. After confirming the straightening of the caudal septum and the widening of the nasal cavity, the hemitransfixion incision was closed with a 4-0 polydioxanone suture. A Rapid Rhino™ was inserted into the nasal cavity and removed the following day.

## Results

Table 1 summarizes the patient demographics and CT data. The MICST group (n=45) comprised 37 males and 8 females, with an average age of 42.2±16.2 years. The CST group (n=45) encompassed 35 males and 10 females, with an average age of 41.0±13.3 years. No significant differences were observed in age or sex between the groups. The mean follow-up period was 12 months (with a maximum of 2 years).

**Table 1:** Patient demographics and results of the computed tomography analysis Patient demographics and CT data were analyzed using the Student's t-test, and the Mann-Whitney test was used to analyze the proportion of cases in which the NT decreased by ≥3 mm with a p-value of <0.05 indicating statistical significance. MICST: minimally invasive cutting and suture technique; CST: cutting and suture technique; VAS: Visual analog scale; NT: nasal tip; CT: computed tomography

Procedure		MICST	CST	Test Statistics	P-value
Patients (n)		45	45		
Age (years)		42.2±13.3	41.0±2.4	t=-0.383	0.603
Sex	Male (%)	35 (77.8)	37 (82.2)	U=693	0.649
	Female (%)	10(22.2)	8 (17.8)		
N/W ratio	Preoperative	0.60±0.19	0.57±0.15	t= 0.982	0.329
	Postoperative	0.80±0.12	0.84±0.13	t=-1.669	0.098
VAS	Preoperative	7.94±1.50	8.26±2.13	t=-0.643	0.172
	Postoperative	1.24±1.40	0.74±0.73	t=-1.385	0.127
NT (mm)	Preoperative	25.2±2.4	24.7±2.3	t=0.797	0.148
	Postoperative	24.6±2.9	23.8±2.4	t=1.450	0.127
NT decrease ≧ 3 mm		n = 0	n = 4	U=1103	0.021
Operative time (min)		40.1±8.6	48.5±7.7	t=4.884	<0.001

**Figure 5:**
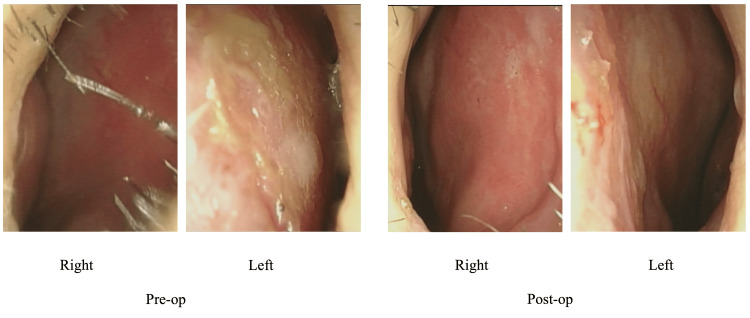
Pre- and postoperative endoscopic findings of a patient undergoing MICST The CED is observed in the left direction preoperatively and resolved postoperatively. MICST: minimally invasive cutting and suture technique; CED: caudal end deviation

**Figure 6:**
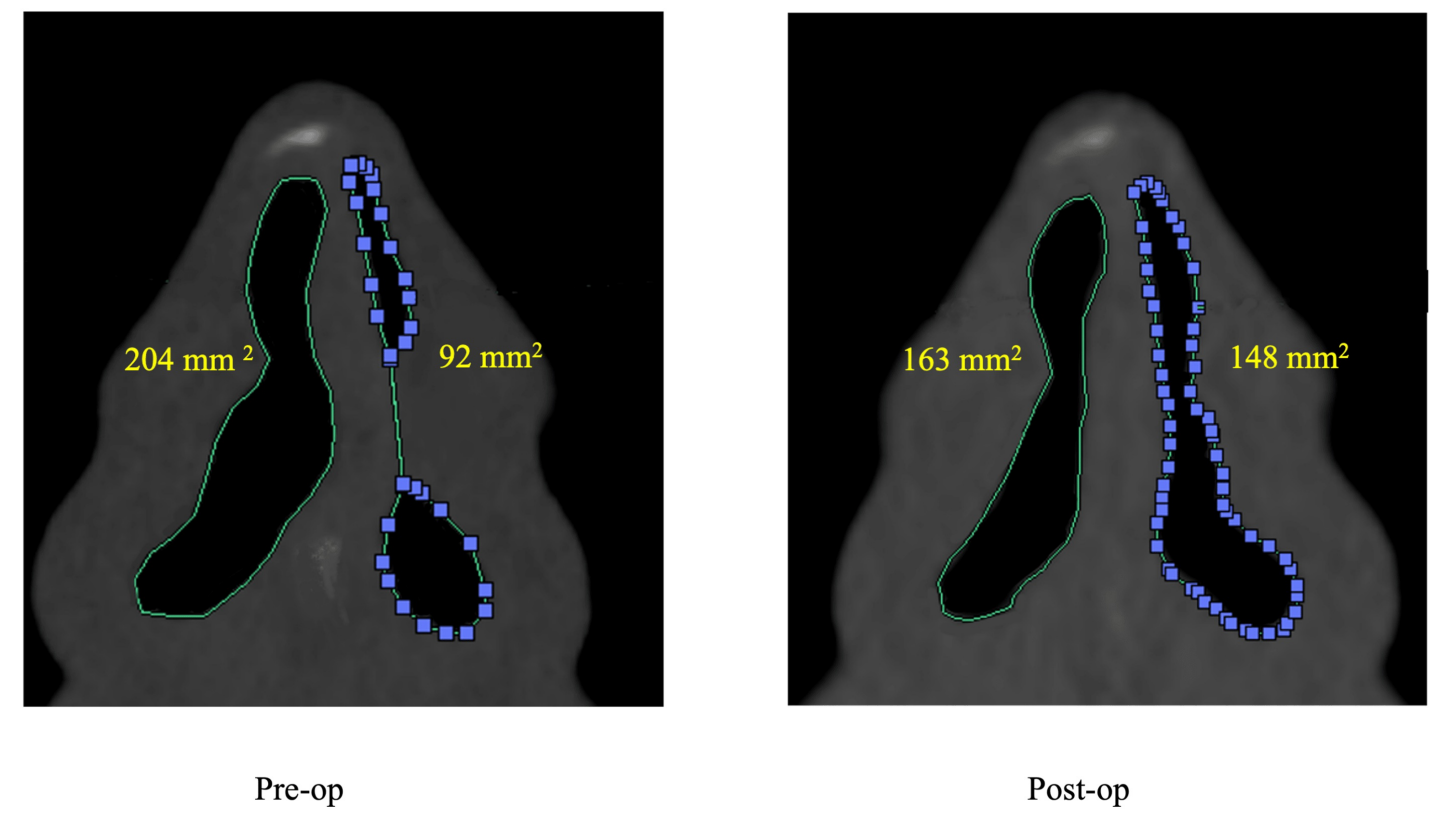
Comparison of the pre- and postoperative comparison N/W ratio following the MICST The change of N/W ratio following the MICST to a 37-year-old male is shown.The pre- and postoperative N/W ratio was 0.45 (92/204) and 0.91 (148/163), respectively. N/W ration: cross-sectional area ratios of the convex side (narrower) and the concave side (wider)

Changes in the N/W ratio and endoscopic findings from a typical case -a 37-year-old male- in which MICST was performed are shown in Figures 5, 6. The N/W ratios were 0.45 (92/204) preoperatively and 0.91 (148/163) postoperatively.

The preoperative/postoperative N/W ratios were 0.60±0.19/0.80±0.12 for the MICST group, and 0.57±0.15/0.84±0.13 for the CST group. Significant differences were noted in both groups between pre- and postoperative values. The degree of increase in the N/W ratio postoperatively was 0.19±0.17 in the MICST group and 0.25±0.17 in the CST group, with no significant differences observed between the groups.

The preoperative/postoperative VAS scores for the MICST were 7.94±1.50/1.24±1.40, and 8.26±2.13/0.74±0.73 for the CST group. Both groups showed significant improvement (p<0.01). The degree of decrease in VAS postoperatively was 6.71±2.11 in the MICST group and 7.52±2.38 in the CST group, with no significant differences noted between the groups.

The preoperative/postoperative NT measurements were 25.2±2.4 mm for the MICST group, and 24.7±2.3 mm/23.8±2.4 mm for the CST group. The degree of decrease in NT postoperatively was 0.6±1.1 mm in the MICST group and 1.0±1.5 mm in the CST group. No significant differences were observed between pre and postoperative data in either group. A decrease in NT of ≥3 mm was observed in 0 (0.0%) of the 45 cases in the MICST group and 4 (8.9%) of the 45 cases in the CST group, showing significant differences between the groups (p<0.05).

Operative time averaged 40.1±8.6 minutes for the MICST group and 48.5±7.7 minutes for the CST group, with significant differences noted (p<0.01).

No postoperative complications such as saddle nose deformity, noticeable NT drop, septal perforation, hematoma, infection, or nasal bleeding were observed during the 6- and 12-month follow-ups at the outpatient clinic for any group.

## Discussion

A deviated nasal septum is a common condition in ENT clinical practice and a primary cause of nasal obstruction. The caudal end forms the medial boundary of the internal nasal valve [8]. Severe nasal obstruction can remarkably interfere with daily activities and may contribute to the etiology of chronic rhinosinusitis [9,10]. In cases of CED, the nasal septal cartilage is misaligned, accounting for approximately 40% of deviated septum cases [5,11]. Even mild CED can cause substantial obstruction as it occurs in the narrowest part of the nasal cavity [12]. Grymer et al. used pre- and postoperative acoustic rhinometry measurements to demonstrate that the impact of nasal obstruction from minimal anterior nasal septum deviations is considerably greater than that from large posterior deviations [13].

Surgery for CED is challenging due to the cartilage's shape memory, the need for adequate nasal tip (NT) and dorsal septal support, and the long-term effects of healing [6]. These factors are crucial for preventing complications, such as cartilaginous dorsal dislocation and columellar collapse, as well as maintaining tip definition and rotation. Inappropriate correction may reduce the height of the NT and shorten the caudal septal length, potentially leading to a saddle nose deformity [3]. Certain surgical techniques, such as the swing door technique and extracorporeal septoplasty, require segmenting the septum from the ANS and reattaching it as part of the procedure [14-16]. To prevent external nasal deformities, it is essential to consider not only preserving the keystone area but also the positional relationship between the PSA and the ANS. The PSA is a crucial landmark during septal surgery. When resecting septal cartilage and the nasal crest, it is important to leave an adequate amount of caudal cartilaginous septum for structural support. Minimal contact between the PSA and the maxillary crest increases the risk of instability and collapse [16]. Additionally, excessive removal of tissue around the ANS can compromise nasal structure by damaging Pitanguy's ligament, which adheres to the ANS [17,18].

Several intranasal approaches that preserve the ANS and PSA have been reported. Jang et al. described the caudal septal trim (CST), which directly treats the caudal end and corrects deviations [3]. An excessively long caudal septum, the primary cause of CED, can be shortened by cutting and overlapping the most convex part of the caudal septum L-strut. While effective, this method can thicken the septum with the addition of a batten graft. Excessive overlap or loosening of sutures may reduce the NT height and shorten the caudal septal length, leading to saddle nose deformity.

There are two surgical procedures reported to be less likely to cause external nasal deformity. The first is Cheon et al.'s partial cutting and suturing technique, which involves an angled incision in the anteroposterior direction at the most curved part of the caudal L-strut and vertical sutures at the middle of the cut edge, then applied to the posterior part of the L-strut [19]. Hosokawa et al. described a modified technique where the septal cartilage is cut about 3 mm above the ANS to create a bank of cartilage and trim excess [7]. The cartilage is repositioned medially after releasing pressure and minimal straightening, and then stitched to the concave side of the nasal septal mucosa on the ANS.

However, these methods are unsuitable for advanced CED cases as they preserve the central portion of the caudal end, which has the strongest curvature [7,19]. Moon et al. have reported surgical procedures that involve incisions both anterior and posterior to the nasal septum cartilage, requiring fixation with polycaprolactone. It is crucial to note that these surgical procedures are not indicated if the PSA is dislocated from the ANS [20].

The MICST is a surgical technique developed from the principles of CST, designed to conservatively maintain the stability of the external nose. This method preserves the tissue around the PSA while maintaining the continuity of the nasal septum cartilage from the keystone area to the anterior nasal spine (ANS). The cutting line is oriented parallel to the dorsal line of the nose, which helps relieve excess pressure from both dorsal and caudal deviations. Additionally, MICST applies to cases with dislocation of the PSA from the ANS, as it does not require preserving the relationship between these structures. As a result, MICST has shown improvements in the N/W ratio and VAS scores, comparable to those achieved with CST, yielding good outcomes, both objectively and in terms of subjective symptoms. Notably, no patients in the MICST group experienced a reduction in NT height of ≥3 mm, whereas four patients in the CST group did. MICST also potentially reduces operative time, with an average duration of 40.1±8.6 minutes compared to 48.5±7.7 minutes for CST. However, the difference of less than 10 minutes suggests limited potential for cost savings and patient benefits.

Our study had some limitations. First, the surgeries were performed during different periods for the MICST and CST groups, and this study did not aim to establish statistical equivalence or discuss the clinical relevance of observed differences, such as changes in nasal tip (NT) height. Second, we did not perform physiological evaluations using acoustic rhinometry. Third, the number of patients was small, and the follow-up period was brief. There is a need to increase the sample size and extend the follow-up duration to better assess the risk of recurrence of external nasal deformity and nasal obstruction. Fourth, the appropriateness of setting the resection margin at 5 mm posterior to the nasal septum cartilage has not been examined. In cases of fragile cartilage, the strength may not be adequately maintained within this range. Conversely, in mild cases of curvature, a wider preservation might be feasible. Fifth, there are limitations to correcting deviations where the upper part of the perpendicular plate of the ethmoid bone is involved, as the septal cartilage cannot be separated from the perpendicular plate in the dorsal strut.

## Conclusions

We report the use of MICST, a novel surgical technique, developed for correcting CED. This method successfully maintains approximately 5 mm of continuity in the posterior nasal cartilage and preserves the connective tissue surrounding the ANS. MICST produced satisfactory results, both subjectively and objectively, comparable to those achieved with the CST. Notably, this was associated with no complications related to external nasal deformities. Furthermore, the improvements in nasal obstruction with MICST were equivalent to those seen with CST while presenting a low risk of external nasal deformity.
